# Editorial: Current advances in the study of Down Syndrome: From development to aging

**DOI:** 10.3389/fnins.2023.1161147

**Published:** 2023-03-20

**Authors:** Aaron Johnstone, William Mobley

**Affiliations:** University of California, San Diego, La Jolla, CA, United States

**Keywords:** Down Syndrome, Alzheimer's disease, trisomy 21, neurodegeneration, neurodevelopment, amyloid precursor protein (APP)

We are pleased to present this Special Research Topic on advancements in modeling both developmental and age-related changes in Down Syndrome, including Alzheimer's disease (AD) in Down syndrome (DS-AD). AD is characterized by a progressive deterioration of memory and other neural functions, resulting in impairments to decision-making, behavior, and sleep; people with DS are at markedly increased risk of AD due to an extra copy of the amyloid precursor protein (*APP*) gene on chromosome 21. Recent decades have witnessed increased life expectancy for people with DS, due to improved patient care, especially repair of developmental cardiac defects. Thus, DS now poses challenges for both the developing and aging brain.

A common theme emerges from the studies collected here: the need to better define the developmental and degenerative alterations caused by trisomy 21 to discover therapies that lessen the impact and enhance the wellbeing of those with DS and their families.

## Modeling AD-DS

### Rodent models

Rodent models of DS have played an important role in revealing genes and mechanisms underlying both developmental and age-related changes. The Ts65Dn mouse, a segmental trisomy due to translocation of a distal region of mouse chromosome 16 to the centromeric region of mouse chromosome 17, contains a large number of mouse genes homologous to those on chromosome 21. Created by Davisson et al. (1993), this was the first truly useful model for examining DS and generated considerable interest in the research community. Indeed, it enabled studies of both developmental and degenerative phenotypes. Klein and Haydar review work in the Ts65Dn mouse focused on intellectual impairments and studies to explicate underlying molecular and cellular mechanisms. While for more than 25 years this model has allowed significant progress to be made, it is susceptible to criticism. One is that phenotypic drift has been observed in some cohorts. Another is that this mouse contains an extra copy of ~60 genes on mouse 17 that are not chromosome 21 homologs. To address the latter criticism, increasingly faithful genetic models have been created using chromosomal engineering. The Dp1Tyb/Dp(16)1Yey mouse models have been used to establish a dose-effect of the *APP* gene in creating DS-AD phenotypes. Additional genes present in excess likely contribute to this condition. Farrell et al. compare rodent models, and their use for *in vivo, ex vivo*, and *in vitro* studies of amyloid-β (Aβ) and neurofibrillary tau dynamics, neuron loss, neuroinflammation, and intracellular signaling and trafficking. Both Farrell et al. and Klein and Haydar look forward to the utility of next-generation humanized rodent models. Of particular interest are MAC21 (mouse) and TcHSA21rat (rat), both of which harbor an artificial chromosome containing the 34 Mb q arm of human chromosome 21. The earliest reports indicate these models recapitulate some behavioral deficits. However, hippocampal volume was unchanged in MAC21 mice verses controls. Given the loss of volume observed in humans and in other mouse models of DS, it remains to be understood which aspects of DS are best modeled by the new humanized lines. More generally, given that no model perfectly replicates the biology of DS, investigators must consider both their benefits and shortcomings with respect to phenotypes and mechanisms of interest.

### Organoid models

Organoids generated from human induced pluripotent stem cell (iPSC) lines have emerged as a complement and alternative to animal models. Li et al. used a pair of iPSC lines—an adult female with DS with her isogenic control—to generate trisomic and control organoids. Using single cell RNA sequencing (scRNA-seq) to examine cell type-specific alterations in transcription, the study found that most dramatically affected by trisomy 21 were a population of excitatory neurons, most closely resembling layer IV cortical neurons. In comparing their findings to other DS transcriptomic datasets, the authors found significant overlaps, but also differences, suggesting that variability can be expected when examining different types of samples from different individuals.

Czerminski et al. argue for caution in interpreting data from experiments using organoids to understand alterations caused by trisomy 21. An initial study found many genome-wide differences in cell type proportions in organoids derived from one trisomic iPSC line, compared to those generated from one disomic line. This motivated an expanded study, which measured ~1,200 organoids from six independent iPSC lines. Surprisingly, while increased expression of trisomic genes was detected, the differences in non-chromosome 21 genes were no longer detectable. These findings indicate that the variability between isogenic lines was greater than the effect of trisomy 21, highlighting the importance of using multiple independent lines.

## Identifying alterations in the human DS brain

Resolving the cellular developmental changes in the human DS brain is complicated by the rarity of postmortem tissue. A meta-analysis of studies of cortical development by Risgaard et al., draws a focus on the lack of well-controlled studies of cortical development in DS and the need for larger sample sizes, especially during perinatal development. The review is a call for action: post-mortem analyses with adequate sampling to allow robust statistical testing are needed to understand changes in cellular composition in perinatal DS.

Impaired neurogenesis during development is a central cause of intellectual impairment in individuals with DS. Intriguingly, a growing body of evidence from animal models supports the plausibility of pharmacological intervention during embryonic and neonatal phases to mitigate deficits in development. Stagni and Bartesaghi review the timeline of neurogenesis to define a treatment window. They then document pharmacological studies carried out in the fetal or neonatal period, most of which employed Ts6Dn mice. Several substances, whose use was supported by an understanding of developmental changes in DS, were shown to rescue or partially rescue defects. The authors appraise the potential for future interventions to enhance neural development in those with DS.

Loss of gray matter volume in DS-AD is well-documented, but a detailed picture of white matter pathology has been slower to emerge. Here, Saini et al. review progress made using magnetic resonance imaging (MRI)-based diffusion techniques to study altered connectivity in DS. Whereas volumetric MRI techniques have broadly revealed a decreased white matter volume in DS, diffusion-based techniques have enabled studies of microstructural integrity at earlier stages in the neurodegenerative process. Convergent findings from these studies revealed a reduction of long association fibers in children and adults with DS, and a reduction in DS-AD vs. individuals with DS but without AD. Reduction of the cingulum bundle was observed not only in adults, but also in young children with DS, suggesting a developmental component to the reduced connectivity of the tract. Reductions were also observed in commissural fibers, including the corpus callosum, and in projection fibers. Taken together, these findings support the use of diffusion imaging techniques to identify biomarkers for early AD detection in DS.

Women in the general population are more likely to be affected by AD than men, but sex-specific effects of DS and AD-DS remain poorly defined. Andrews et al. review the variable findings that have been reported to date; while several studies have indicated that women with DS have higher risk of developing dementia, others have found no (or opposite) effect of sex. The authors discuss the need to control for hormone changes, as women with DS experience earlier onset of menopause and concomitant reduction in estrogen than women without DS. A growing body of work points to a role for estrogen in healthy brain aging, though studies of hormone replacement therapy in general population sporadic AD have been inconclusive.

Taken together, these articles form a unifying theme: *more and better* experimental controls, sample sizes, and models are needed to enable much-needed progress in understanding DS and DS-AD.

## Author contributions

AJ participated in preparing the text of the editorial. Both authors contributed to the article and approved the submitted version.
